# Adjuvant therapy for locally advanced renal cell cancer: A systematic review with meta-analysis

**DOI:** 10.1186/1471-2407-11-115

**Published:** 2011-03-31

**Authors:** Adolfo JO Scherr, Joao Paulo SN Lima, Emma C Sasse, Carmen SP Lima, André D Sasse

**Affiliations:** 1 CEVON - Centro de Evidências em Oncologia UNICAMP - Universidade Estadual de Campinas, São Paulo, Brazil

**Keywords:** Antineoplastic agents, drug therapy, renal cell carcinoma, meta-analysis

## Abstract

**Background:**

Many adjuvant trials have been undertaken in an attempt to reduce the risk of recurrence among patients who undergo surgical resection for locally advanced renal cancer. However, no clear benefit has been identified to date. This systematic review was conducted to examine the exact role of adjuvant therapy in renal cancer setting.

**Methods:**

Randomized controlled trials were searched comparing adjuvant therapy (chemotherapy, vaccine, immunotherapy, biochemotherapy) versus no active treatment after surgery among renal cell cancer patients. Outcomes were overall survival (OS), disease-free survival (DFS), and severe toxicities. Risk ratios (RR), hazard ratios (HR) and 95% confidence intervals were calculated using a fixed-effects meta-analysis. Heterogeneity was measured by I^2^. Different strategies of adjuvant treatment were evaluated separately.

**Results:**

Ten studies (2,609 patients) were included. Adjuvant therapy provided no benefits in terms of OS (HR 1.07; 95%CI 0.89 to 1.28; P = 0.48 I^2 ^= 0%) or DFS (HR 1.03; 95%CI 0.87 to 1.21; P = 0.77 I^2 ^= 15%) when compared to no treatment. No subgroup analysis (immunotherapy, vaccines, biochemotherapy and hormone therapy) had relevant results. Toxicity evaluation depicted a significantly higher frequency of serious adverse events in the adjuvant group.

**Conclusions:**

This analysis provided no support for the hypothesis that the agents studied provide any clinical benefit for renal cancer patients although they increase the risk of toxic effects. Randomized trials are underway to test targeted therapies, which might open a new therapeutic frontier. Until these trials yield results, no adjuvant therapy can be recommended for patients who undergo surgical resection for renal cell cancer.

## Background

Renal cancer is among the tenth most common cancers and its incidence has increased constantly in recent decades[1]. Two thirds of patients have no evidence of distant metastasis at diagnosis, and radical surgery can be curative. However, just a fraction of these patients are effectively cured by surgery as recurrence occurs in a high proportion of cases[2].

In the last 30 years, only a few drugs have shown some activity against advanced renal cancer. Initially immunomodulators, namely, interferon and interleukin-2, were used to control metastatic disease and, in unpredictable instances, could stabilize the disease for years or even eliminate it completely[3,4].

The existence of rare but exceptional results with immunomodulators in metastatic patients triggered initiation of trials testing these drugs, combined or not with antineoplastic agents in the adjuvant setting. Some trials tested immunotherapy (interleukin or interferon) or vaccines derived from tumor cells. Conventional antineoplastic agents or hormonal therapy were also tested[5,6]. Unfortunately, such therapies have not shown clear evidence of survival improvement to date[7,8]. Targeted therapies are already being tested in the adjuvant setting, however no mature survival data are currently available.

In this scenario, we carried out a systematic review with meta-analysis of randomized trials to address the efficacy of adjuvant therapy among patients who undergo surgical resection for renal cell cancer.

## Methods

The present systematic review was originally completed in the context of an evidence-based training, based on the Centre of Evidences in Oncology (CEVON) workgroup, in the State University of Campinas (UNICAMP), Brazil. All the evidences were selected and reviewed by two members of CEVON and discussed with the group and the coordinator (ADS). All work produced by CEVON is editorially independent and does not have any funding source.

### Search strategy

Studies were searched and identified in electronic databases (PubMed/MEDLINE, EMBASE, LILACS, ClinicalTrials.gov and The Cochrane Library). Websites for ASCO, AUA, ECCO and ESMO meetings were also scrutinized. We used a sensitive search strategy with words related to kidney (kidney OR renal), cancer (tumor OR neoplasm OR carcinoma OR cancer), adjuvant therapy (chemotherapy OR drug therapy OR immunotherapy OR biotherapy OR hormone*), and randomized trials (random* OR randomized trials) in all fields. The search was restricted to trials published or presented in English.

We hand-searched the reference lists of related reviews for additional publications. All references of relevant articles were scanned and all additional studies of potential interest were retrieved for further analysis. The search included literature published or presented until June 2010.

### Selection criteria

We sought to identify all published or presented randomized controlled clinical trials comparing post-surgical therapy versus no further active therapy (placebo or observation) in patients who underwent surgery for renal cell cancer. Eligible trials included patients with renal cell cancer of any histological type, with no sign of metastases and rendered disease free after radical surgery. Trials enrolling patients with metastatic and non-metastatic disease were included if separate information on non-metastatic patients was provided. Trials involving radiation as adjuvant therapy were excluded.

The original published articles of all relevant citations were retrieved for a more detailed analysis. No attempt was made to restrict the search according to more specific methodological characteristics.

Two reviewers (AJOS and ADS) analyzed the list of references and independently selected the studies. The final selection of which studies to include was achieved by consensus.

### Data extraction

The name of the first author and the year of publication of the article were used for identification purposes. Two reviewers (AJOS and ADS) independently extracted the data from the studies. A third reviewer (CSPL) was consulted to solve disagreements.

The primary outcome analyzed was overall survival (OS). Other endpoints of interest were disease-free survival (DFS), and the incidence of Common Toxicity Criteria (CTC) scale grade 3/4 toxicities. When the published article did not present needed data to determine OS or DFS, the authors were contacted to provide the information. Toxicity data were retrieved, as available, in the publication.

The hazard ratios (HRs) of time-to-event data (OS and DFS) were directly extracted from the original study or were estimated indirectly using either the reported number of events and the corresponding P value for the log-rank statistics, or by reading off survival curves, as suggested by Parmar and colleagues[9]. The calculations were carried out using the spreadsheet provided by Tierney and colleagues[10]. The number of events and number under risk were abstracted for toxicity comparison.

### Statistical analysis and synthesis

Details regarding the main methodological dimensions empirically related to bias[11] were extracted, and the methodological quality of each selected trial was assessed by two reviewers (AJOS and ADS). Special attention was given to the generation and concealment of the sequence of randomization, blinding, whether an intention-to-treat analysis was performed or not, use of placebo, and source of funding. These data were applied in a subgroup, and sensitivity analyses were performed to test the stability of our conclusions.

All meta-analyses were performed using Review Manager 5 (RevMan 5; The Nordic Cochrane Centre, The Cochrane Collaboration, Copenhagen, Denmark) with a fixed-effect model. Time-to-event outcomes were compared using HR while an odds ratio (OR) was used for toxicity evaluation. The effect of the treatment was expressed as a ratio of active therapy arm over the placebo/observation arm. Thus, in OS and DFS evaluations, an HR value less than 1 favored active therapy, whereas an HR greater than 1 favored observation. Respective 95% confidence intervals (CI) were calculated for each estimate.

In the safety analyses, an OR less than 1 favored active therapy while an OR greater than 1 favored observation. The number needed to harm (NNH) for risks, derived from the inverse of the absolute risk difference, was also used to measure toxicity risk.

Statistical heterogeneity of the results of the trials was assessed by the chi-square (χ^2^) test[12], and was expressed as the I^*2 *^index, as described by Higgins and colleagues[13]. When a considerable heterogeneity was detected (I^*2 *^>40%), a possible explanation for it was pursued. When a reasonable cause was found, then a separate analysis was performed. If the cause was not apparent and heterogeneity was caused by divergent data in terms of direction of results (i.e., data favoring one or other treatment), we chose not to pool the data. Publication bias was evaluated by Egger's test[11].

All different therapies - hormonal, biochemotherapy, chemotherapy, vaccine, and immunotherapy - were analyzed separately to access their impact in survival and safety.

## Results

### Literature Search

**Figure 1:**
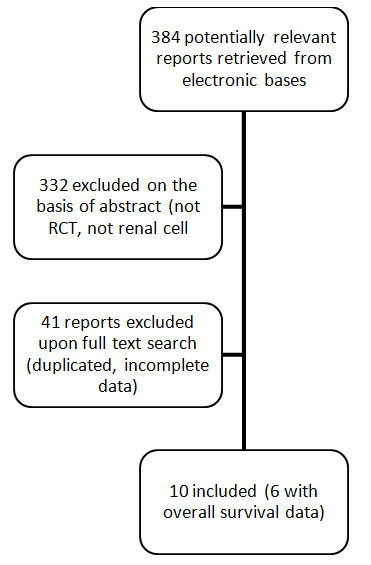
**QUOROM flowchart of the systematic literature review**. Abbreviations: RCT: randomized controlled trial.

The systematic search is summarized in the QUOROM flowchart (Figure 1). Twelve trials were identified that were published or presented between 1987 and 2009[5-8,14-21]. Two studies enrolled metastatic and non-metastatic patients but no separate information of non-metastatic was provided, which precluded their inclusion in analyses[8,15]. The remaining ten trials comprised 2609 patients. Six trials, (1,997 patients) had mature OS data [7,14,16,18-20] while DFS was reported in all studies.

Eligible trials enrolled high-risk patients. Approximately 60% of patients had lymph node positive disease while 86% had pT2 or more advanced disease. No patient had previously received systemic therapy. None of selected trials was a placebo-controlled, double-blind trial. The Wood trial demanded a minimum clear cells component (25%) in tumor burden[19]. The remaining trials accepted all pathological subtypes. Considering the selected studies, three were carried out in the United States, six in Europe, and one in Japan. Methodological details potentially related to bias are described in Table 1.

Three studies tested vaccine therapy[16,17,19], three interleukin/interferon therapy[7,18,20] without high dose therapy, one biochemotherapy[14], one hormone therapy[5], one thalidomide[21] and one chemotherapy alone[6]. A detailed description of treatment arms for all included studies is presented in Table 2.

### Overall Survival

The impact of adjuvant treatment on OS was extracted directly or estimated indirectly from published data of six trials with mature data (1,997 patients)[7,14,16,18-20]. No single study demonstrated a statistically significant improvement in OS. Funnel plots of all comparisons did not identify a publication bias.

As the trials whose results were analyzed involved the use a multitude of agents, some of them with limited activity in advanced disease, the subgroups are shown and described individually.

#### Vaccine therapy

**Figure 2:**
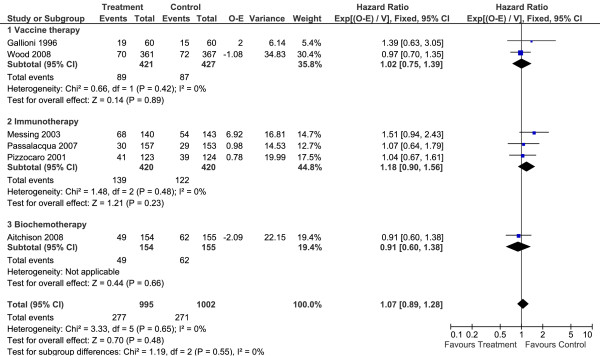
Overall survival meta-analysis of adjuvant therapies versus no therapy

Two trials identified (848 patients) provided OS data on vaccine therapy[16,19]. Meta-analysis demonstrated that adjuvant therapy was not capable of improving OS (HR = 1.02; 95% CI 0.75 to 1.39; P = 0.89; Figure 2). There was no heterogeneity between trials (I^2 ^= 0%).

#### Immunotherapy

Three trials (840 patients) with immunotherapy were gathered and there was no sign of OS improvement (HR = 1.18; 95% CI 0.90 to 1.56; P = 0.23; Figure 2)[7,18,20]. Again, no heterogeneity was found (I^2 ^= 0%).

#### Other Therapies

The systematic review found only one trial testing biochemotherapy (5-fluorouracil associated with interferon alfa/interleukin 2)[14]. There was no survival gain with biochemotherapy (HR = 0.91; 95% CI 0.60 to 1.38; P = 0.66; Figure 2).

One study tested chemotherapy (UFT), one thalidomide, and another one hormone therapy (medroxiprogesterone)[5,6,21]. None presented OS data.

The meta-analysis of all studies demonstrated that the agents studied did not improved OS (HR = 1.07; 95%CI 0.89 to 1.28; P = 0.46; Figure 2). There was no heterogeneity between trials (I^2 ^= 0%; P = 0.64).

### Disease-free Survival

Information concerning DFS was available in all trials (ten trials, 2,609 patients). Only one study demonstrated a statistically significant result, favoring active therapy[17].

One more time, as the trials used many different agents, some of them with no activity in advanced disease, the subgroups are shown and described individually.

#### Vaccine Therapy

All three trials identified testing vaccines presented DFS data (1,227 patients)[16,17,19]. The meta-analysis could not identify a DFS gain (HR = 0.86; 95% CI 0.71 to 1.04; P = 0.13; I^2 ^= 51%) (data not shown). Nevertheless an elevated heterogeneity was found that demanded a more detailed evaluation of this comparison.

**Figure 3:**
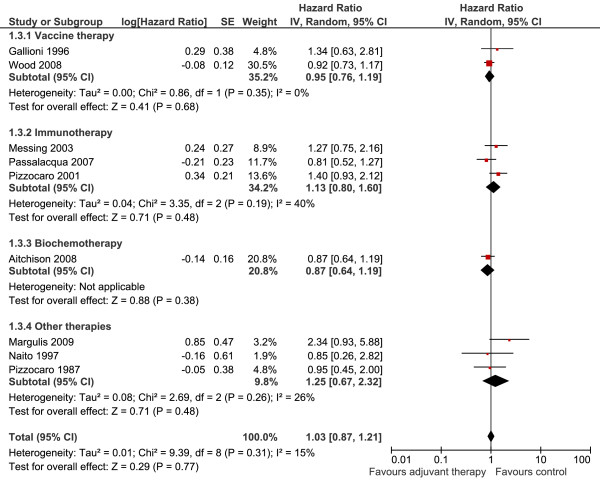
Disease-free survival meta-analysis of adjuvant therapy versus no therapy

Examining carefully the characteristics of each trial, the study conducted by Jocham et al[17] seemed to be the source of heterogeneity. Jocham et al applied autologous vaccine in radically resected renal cancer patients and was the unique trial identified with positive impact in survival, more specifically, DFS. However, this study had methodological restrictions. A large portion of patients (41%) were not properly followed due to histological incompatibilities, lost of follow-up, failure in vaccine production, and staging flaws. Taking all these into account, Jocham et al results must be viewed with great caution. Excluding this trial from analysis (848 patients left) meta-analysis did not identify a DFS gain while heterogeneity was eliminated (HR = 0.95; 95% CI 0.76 to 1.19; P = 0.68; I^2 ^= 0%; Figure 3).

#### Immunotherapy

All three trials (840 patients) with immunotherapy provided DFS data[7,18,20]. The meta-analysis was performed with acceptable heterogeneity although no gain could be observed (HR = 1.13; 95% CI 0.80 to 1.60; P = 0.48; I^2 ^= 40%; Figure 3).

#### Other Therapies

The situation of thalidomide, chemotherapy, hormone therapy, and biotherapy was identical: the systematic review identified just one trial testing each of these therapies and no study demonstrated a survival benefit of adjuvant treatment.

Naito[6] tested adjuvant UFT (HR = 0.85; 95% CI 0.26 to 2.82; P = 0.80); Pizzocaro[5] tested medroxiprogesterone (HR = 0.95; 95% CI 0.45 to 2.00; P = 0.90); Margulis[21] tested thalidomide (HR = 2.34; 95% CI 0.93 to 5.88; P = 0.07), and Aitchison[14] applied 5-fluorouracil and interferon alfa/interleukin 2 (HR = 0.87; 95% CI 0.64 to 1.19; P = 0.40).

The meta-analysis of all studies demonstrated that all agents studied did not improved DFS (HR = 1.03; 95%CI 0.87 to 1.21; P = 0.77) (Figure 3). There was no significant heterogeneity between trials (I2 = 15%; P = 0.31).

### Toxicity

Eight trials presented toxicity data (1,910 patients)[5-7,16,17,19,20]. Adverse event descriptions were scarce and no single toxicity was described across all trials. Vaccine and immunotherapy caused mild but frequent skin induration, injection site pain, and flu-like symptoms. Just one trial, which tested vaccine therapy, described grade 3/4 neutropenia (RR = 62.33; P = 0.004) and anemia (RR = 3.06; P = 0.49)[7]. No trial described neutropenic fever, thrombocytopenia, or grade 5 events.

Despite the absence of details, most severe toxicities were presented in each trial and therefore worst toxicity meta-analysis was feasible. Table 3 depicts the number of grade 3/4 events among the patients at risk (safety population).

## Discussion

This systematic review sought to identify all types of drug interventions used as post-surgical therapy for resected renal cell cancer. The evidence indicates that the adjuvant approaches studied are not capable of improving survival of non-metastatic renal cancer patients while exposing patients to unnecessary toxicity.

We included a broad spectrum of interventions to be evaluated in this meta-analysis - immunotherapy, antiangiogenic, hormonal and cytotoxic drugs combined or not with immunotherapy. None were shown to be effective in what could be interpreted as an absence of activity for each one of these approaches in the adjuvant setting.

Among the included trials, one deserves specific attention. Jocham et al[17] tested an autologous vaccine and was the only trial to present a positive DFS result. However, these results might have been compromised due to worrisome methodological issues discussed here and elsewhere[22]. All this justified the exclusion of the Jocham trial from DFS analysis.

The paucity of adverse event descriptions hampered toxicity meta-analyses and many important events such as febrile neutropenia, nausea, and hypotension could not be evaluated. Even with incomplete reports, a worst toxicity analysis clearly revealed the low tolerability of these therapies.

The present study has the typical limitations and strengths of an aggregated data meta-analysis. We found no indication of such bias using statistical methods designed to detect it. An analysis of individual patient data would be more powerful to address this issue. However it is hardly believable that an individual patient data meta-analysis could be justified after the results of this meta-analysis.

Recently accumulated data in the metastatic setting indicates targeted therapies as the logical option to be tested in adjuvant therapy. These drugs - sorafenib, sunitinib - are already being tested in adjuvant randomized trials (S-TRAC: sunitinib treatment of renal adjuvant cancer; SORCE: sorafenib in treating patients at risk of relapse after undergoing surgery to remove kidney cancer and ASSURE: adjuvant sorafenib or sunitinib for unfavourable renal carcinoma). Trial results will be available between 2012 and 2016[23].

## Conclusions

This systematic review strengthens the evidence that no studied systemic therapy provides improvement in survival for patients who undergo surgical resection of renal cell cancer. Results of targeted therapies in the adjuvant context must be closely observed as they might represent an important shift in the prognosis of resected renal cancer patients.

## Competing interests

The authors declare that they have no competing interests.

## Authors' contributions

AJOS conceived the study, searched databases, selected the trials and extracted the data. JPSNL searched databases, selected the trials, performed the statistical analysis and drafted the manuscript. ECS participated in the selection of trials and extraction of data. CSPL participated in its design and provided administrative support. ADS conceived of the study together with AJOS, participated in its design, statistical analysis and coordination. All authors read and approved the final manuscript

## Pre-publication history

The pre-publication history for this paper can be accessed here:

http://www.biomedcentral.com/1471-2407/11/115/prepub
